# Impaired formation of homotypic cell-in-cell structures in human tumor cells lacking alpha-catenin expression

**DOI:** 10.1038/srep12223

**Published:** 2015-07-20

**Authors:** Manna Wang, Xiangkai Ning, Ang Chen, Hongyan Huang, Chao Ni, Changxi Zhou, Kaitao Yu, Sanchun Lan, Qiwei Wang, Shichong Li, Hong Liu, Xiaoning Wang, Zhaolie Chen, Li Ma, Qiang Sun

**Affiliations:** 1 Institute of Molecular Immunology, School of Biotechnology, Southern Medical University, Guangzhou 510515, P. R. China; 2 Laboratory of Cell Engineering, Institute of Biotechnology, 20 Dongda Street, Beijing 100071, P. R. China; 3 National Key Laboratory of Medical Immunology and Institute of Immunology, Second Military Medical University, 800 Xiangyin Road, Shanghai 200433, P. R. China; 4 Department of Oncology, Beijing Shijitan Hospital of Capital Medical University, 10 TIEYI Road, Beijing 100038, P. R. China; 5 Department of Stomatology, Affiliated Hospital of Academy of Military Medical Science, 8 Dongda Street, Beijing 100071, P. R. China; 6 The Institute of Life Sciences, the State Key Laboratory of Kidney; the Key Laboratory of Normal Aging & Geriatric, the Chinese PLA General Hospital, Beijing 100853, P. R. China

## Abstract

Although cell-in-cell structures (CICs) could be detected in a wide range of human tumors, homotypic CICs formed between tumor cells occur at low rate for most of them. We recently reported that tumor cells lacking expression of E- and P-cadherin were incapable of forming homotypic CICs by entosis, and re-expression of E- or P-cadherin was sufficient to induce CICs formation in these tumor cells. In this work, we found that homotypic CICs formation was impaired in some tumor cells expressing high level of E-cadherin due to loss expression of alpha-catenin (α-catenin), a molecular linker between cadherin-mediated adherens junctions and F-actin. Expression of α-catenin in these tumor cells restored cell-cell adhesion and promoted CICs formation in a ROCK kinase-dependent way. Thus, our work identified α-catenin as another molecule in addition to E- and P-cadherin that were targeted to inactivate homotypic CICs formation in human tumor cells.

Majority of internal lumens in our bodies are covered with a layer of epithelial cells, whose integrity is critical for the organs to function properly. The integrity of epithelial tissues depends on intact adherens junctions (AJs), which is a multiple-components complex comprising cadherins, the transmembrane adhesion receptors, and their cytoplasmic binding proteins such as p120-catenin and β-catenin etc.^1^. Functional AJs is coupled with actin filaments through linker molecules, of which α-catenin and EPLIN are best characterized^2^,^3^. Actin polymerization and actomyosin contraction regulated by Rho GTPases and their effectors play important role in AJs maintenance and remodeling^1^,^4^. Aberrations, structural or functional, in AJs were associated with a number of pathological conditions, such as infection, inflammation and tumors and the like^5^,^6^,^7^. Recent studies indicated that AJs mediated the formation of cell-in-cell structures (CICs)^8^,^9^.

CICs refer to the cellular structures formed between viable cells, in which one or more cells exist inside other ones. Early records on CICs could be dated back to last century, when pathologists identified this type of unusual structures in human tumor samples^10^. Recent progress showed that cell-in-cell structures are rather complex than initially described, and could be classified into homotypic or heterotypic CICs based on the cells involved^10^,^11^. Heterotypic CICs are usually formed by penetration of lymphocytes into tumor cells through processes like emperitosis^12^. Homotypic CICs are formed between cells from same type, for example, epithelial cells inside epithelial cells. Mechanisms like entosis and homotypic cell cannibalism (HoCC) are responsible for this type of CICs formation^8^,^13^. Once formed, CICs usually result in death of the internalized cells, which lead to the conception that CICs formation is a process of cell death^8^. Limited researches identified extensive involvement of CICs in several important biological processes including development, immune homeostasis and tumor development and evolution etc.^11^,^14^.

Recently, we and others found that formation of homotypic CICs by entosis was dependent on intact AJs and polarized actomyosin contraction^8^,^9^,^15^,^16^. Tumor cells lacking epithelial cadherins (E- and P-cadherin) failed to form CICs, moreover, re-expression of E- or P-cadherin could efficiently induce CICs in these cells, suggesting that disrupting AJs is a mechanism whereby tumor cells escape entosis-mediated CICs formation^9^. In this work, we found that tumor cells deficient of α-catenin, a key component of functional AJs, also displayed impaired CICs formation, which could be fixed by restored expression of α-catenin. Therefore, tumor cells could escape entotic CICs formation by targeting multiple AJs components including E-/P-cadherin and α-catenin, and CICs formation by entosis may constitute a novel mechanism underlying the tumor suppressive function imposed by α-catenin.

## Results

### Tumor cells lacking expression of α-catenin show impaired CICs formation

**Figure 1:**
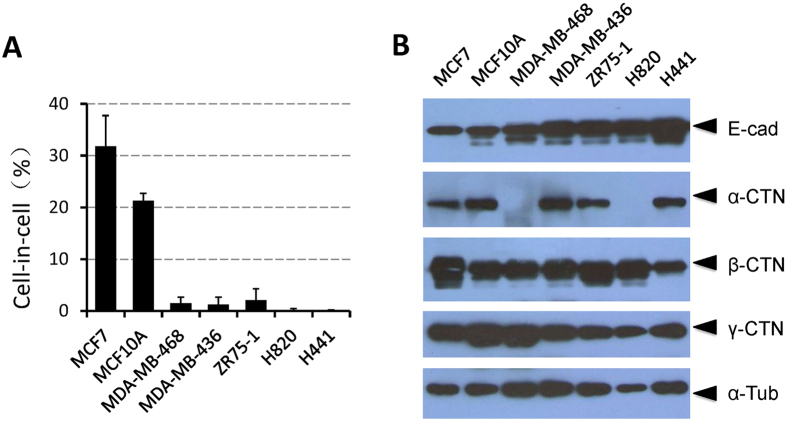
Tumor cells lacking expression of α-catenin show impaired CICs formation. (**A**) Cell-in-cell formation frequencies of tumor cell lines. Cells were suspended for 6 h before analysis. MCF10A is a mammary epithelial cell line. H820 and H441 are lung carcinoma cell lines, the rest are breast cancer cell lines. Data are mean ± SD of triplicate experiments, n > 300 for each cell line. (**B**) Expression of adhesion molecules as detected by western blot.

**Figure 2:**
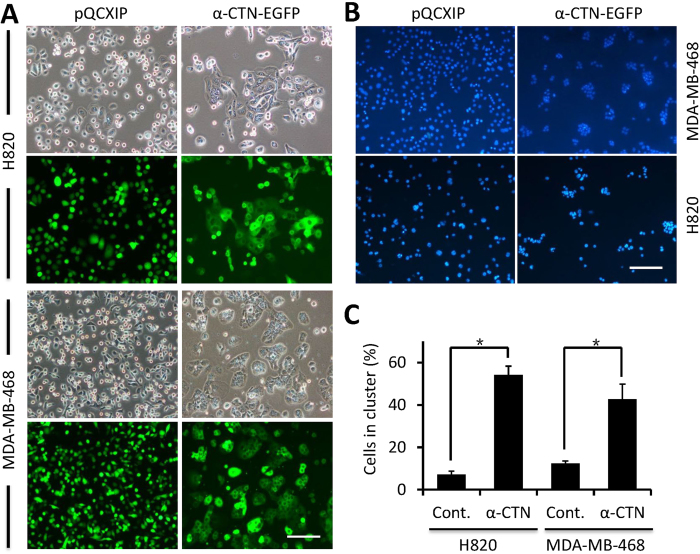
Restoring cell-cell adhesion upon of α-catenin expression. (**A**) Exogenous α-catenin expression induces enhanced cell-cell adhesion and cell clustering in adherent MDA-MB-468 and H820 cells. Scale bars: 50 μm. (**B**) Expression of α-catenin promotes cell clustering in suspension culture. Blue spots show the distribution of the nuclei stained with DAPI. Scale bars: 50 μm. (**C**) The percentage of cells involved in cell cluster. Cells in cluster rate (%) = (number of tumor cells involved in cell cluster / number of total tumor cells counted) ×100. Cell cluster was defined as a cell colony which contains more than 6 cells. Note, pQCXIP refers to empty vector. Data are mean ± SD of cells analyzed in triplicate, n > 300 for each experiment.

In our previous work, we found that loss of E- and P-cadherin caused defective CICs formation in a group of human breast cancer cells, such as MDA-MB-231, MDA-MB-453 and SKBR3 and the like, re-expression of E- or P-cadherin alone was sufficient to induce entotic CICs in these cells^9^. However, we also found that some cancer cells such as MDA-MB-468, although expressed E-cadherin at levels comparable to that of MCF10A, displayed impaired CICs formation. Further investigation indicated that this was also true for some other breast cancer cell lines like ZR75-1 and lung cancer cell lines such as H820 and H441 as well (Fig. 1A,B). Moreover, E-cadherin levels in ZR75-1, H820 and H441 cells are even higher than that in MCF10A and MCF7, two cells show high level of CICs formation upon induction (Fig. 1B), which suggests that mechanisms other than loss of epithelial cadherins should be responsible for defects in CICs formation in these cells. Interestingly, we found α-catenin did not express in two of these cell lines, MDA-MB-468 and H820. Since α-catenin is a functional component of AJs, we therefore hypothesize that loss expression of α-catenin compromised AJs and subsequently CICs formation. In agreement with this idea, we found that cultured MDA-MB-468 and H820 cells displayed a scattered morphology (Fig. 2A), indicating defective cell-cell adhesion.

### Restored cell-cell adhesion upon α-catenin expression

To examine a causal role of α-catenin expression in defective cell-cell adhesion of MDA-MB-468 and H820 cells, we expressed mouse α-catenin tagged with EGFP in these cells. As shown in Fig. 2A, for both MDA-MB-468 and H820 cells, while EGFP-expressing control cells are scattered and loosely interact with each other, cells expressing α-catenin stay together in a compact fashion, indicating that cell-cell adhesion is restored. To confirm this result, we performed the cell cluster assay, a semi-quantitative method to examine cell-cell adhesion. As shown in Fig. 2B,C, more than 50% H820 and 42% MDA-MB-468 cells form clusters composing of >6 cells upon α-catenin expression, which is significantly higher than that in EGFP-expressing control cells (*p* < 0.05). Thus, loss expression of α-catenin is responsible for disrupted cell-cell adhesion mediated by AJs in MDA-MB-468 and H820 cells.

### Expression of α-catenin induces CICs formation

**Figure 3:**
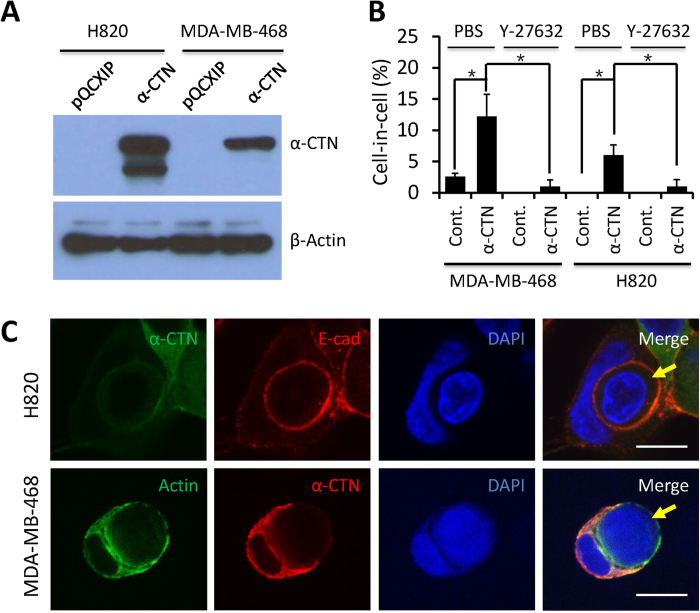
Expression of α-catenin induces CICs formation. (**A**) Western blot shows α-catenin expression in MDA-MB-468 and H820 cells. (**B**) CICs formation in MDA-MB-468 and H820 cells after exogenous α-catenin expression. Cells were cultured for 36 h in suspension in the presence or absence of Y27632, which is an entosis inhibitor. Experiments were carried out in triplicate and the data are presented as mean ± SD. (**C**) Typical cell-in-cell structure in MDA-MB-468 and H820 cells with α-catenin expression. Arrows indicate internalized cells. Scale bars: 10 μm.

We next examine CICs formation in MDA-MB-468 and H820 cells with restored cell-cell adhesion. As evidenced by western blot (Fig. 3A) and EGFP expression (Fig. 2A,C), α-catenin was successfully expressed in these two cells. Upon suspension, CICs were readily detected in both cell lines (Fig. 3B). Importantly, formation of CICs could be significantly inhibited by ROCK kinase inhibitor-Y27632 (Fig. 3B), suggesting that entosis mediates CICs formation in this case. It should be noted that CICs formation could be detected in control MDA-MB-468 cells at low frequency of 2.6% or so (Fig. 3B), suggesting that either mechanisms other than entosis are responsible for formation of these CICs, or some other molecules could partially substitute α-catenin in MDA-MB-468 cells. Mechanistic investigation comparing the differences between MDA-MB-468 and H820 cells will shed light on this issue.

### Depletion of α-catenin compromised cell-cell adhesion and CICs formation

**Figure 4:**
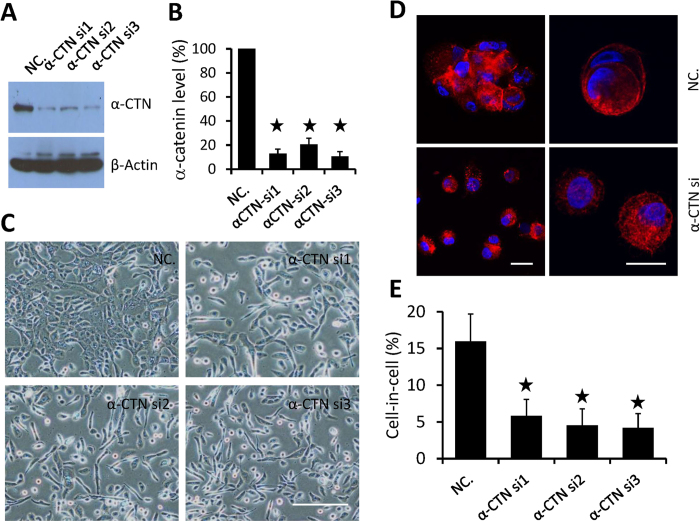
Compromised CICs formation upon α-catenin depletion. (**A**) Western blot shows α-catenin knockdown by siRNAs in MCF10A cells. (**B**) Silencing efficiency of α-catenin in MCF-10A quantified by ImageJ software (NIH). Data are mean ± SD and representative of three independent experiments. (**C**) Compromised cell-cell adhesion in MCF10A cells with α-catenin depleted by siRNAs. Scale bars: 50 μm. (**D**) Representative images of CICs in MCF10A cells. Cells were stained with antibodies against E-cadherin (red), and DAPI (blue) to show nuclei. Scale bars: 10 μm. (**E**) A significant reduction of cell-in-cell formation in MCF10A cells upon α-catenin knockdown. CICs were quantified after 6 h suspension. Data are mean ± SD of triplicate experiments, n > 300 for each experiment.

To further confirm the role of α-catenin in CICs formation, we depleted α-catenin expression by RNA interference in MCF10A cells, which display high frequency of CICs. Transfection of three siRNA duplexes efficiently knocked down α-catenin as detected by Western Blot (Fig. 4A), with silencing efficiency of more than 80% for all three duplexes (Fig. 4B). Expectedly, cell-cell adhesions were disrupted as evidenced by loose cell-cell interaction and scattered morphology even when cultured in high density (Fig. 4C). Meanwhile, CICs formation was consistently inhibited in three siRNAs-transfected cells (Fig. 4D,E). So expression of α-catenin, like E- and P-cadherin, is prerequisite for CICs formation of epithelium-originated cells.

### Remodeled cytoskeleton in alpha-catenin-expressing cells

**Figure 5:**
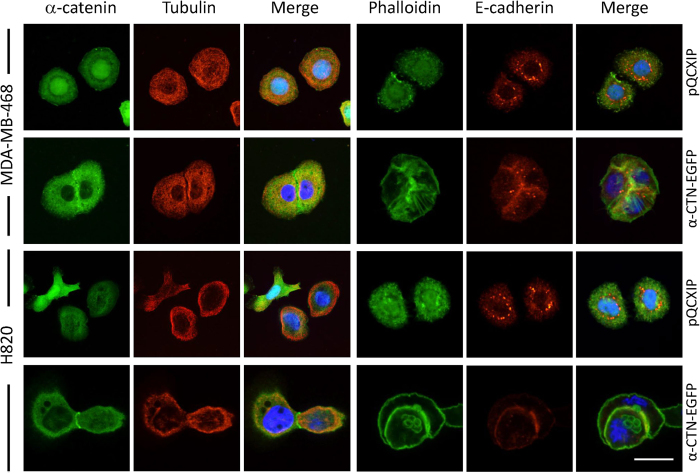
Distribution of AJs and cytoskeleton in α-catenin-expressing tumor cells. Immunostaining of adhesion molecules and cytoskeleton elements as indicated in control and α-catenin-expressing MDA-MB-468 and H820 cells. Scale bars: 20 μm.

**Figure 6:**
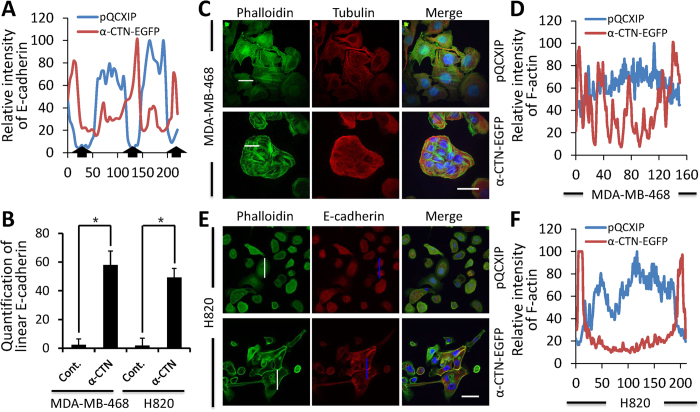
Enhanced F-actin in α-catenin-expressing tumor cells. (**A**) Relative pixel intensities of red fluorescence (E-cadherin) for H820 cells in Fig. 6E by line-scans profiling (blue lines). Black arrows indicate intercellular junctional regions. (**B**) Quantification of intercellular linear E-cadherin upon restored α-catenin expression in MDA-MB-468 and H820 cells. Neighboring cells with linear E-cadherin peak at cell-cell contact was counted as positive, the others were counted as negative. Data are mean ± SD of cell pairs analyzed in triplicate, n > 300 for each experiment, *statistically significant (p < 0.05). (**C**, **E**) Fluorescence images of control and α-catenin-expressing MDA-MB-468 and H820 cells. MDA-MB-468 cells were stained with phalloidin (green) and tubulin (red), and H820 cells were stained with phalloidin (green) and E-cadherin (red). Scale bars: 20 μm. (**D**, **F**) Relative pixel intensities of green fluorescence (F-actin) by line-scan profiling (white lines) (ImageJ software).

Our previous work showed that cytoskeleton was remodeled during CICs formation induced by E-/P-cadherin expression, we therefore examined the distribution of AJs molecules and cytoskeleton upon α-catenin expression. For both adherent MDA-MB-468 and H820 cells expressing α-catenin, E-cadherin locates neatly at the cell-cell boundary, where α-catenin was also enriched (Fig. 5). This pattern of E-cadherin is in sharp contrast to that in control cells, where few linear E-cadherin locates at cell-cell contact region as demonstrated by line-scan analysis (Fig. 6A) and overall quantification (Fig. 6B). Meanwhile, actin filaments seem to be enhanced with more phalloidin-positive filaments detected in α-catenin-expressing MDA-MB-468 cells (Figs 5 and 6C), whereas F-actin distribution displayed less complexity in control cells as demonstrated by pixel intensity profiling (Fig. 6D), which is consistent with α-catenin’s role in bundling actin filaments^17^. The phenotype of enhanced F-actin bundling was even stronger in α-catenin-expressing H820 cells, where F-actin seems increased excessively and almost centralized on the cell cortex (Fig. 6E,F).

## Discussion

Although being extensively reported in human tumors for a long time, the mechanisms underlying CICs formation remained unexplored until recent years, when several research models were established^8^,^12^,^13^. Meanwhile, the research methods were also being developed for both homotypic and heterotypic CICs^18^,^19^, and for subtyping CICs in human tumor samples^20^. Currently, it is well accepted that cell-cell adhesion and cytoskeleton are two key players in CICs formation. Accordingly, a number of molecules were identified belonging to these two categories, including E- and P-cadherin^8^,^9^, Ezrin, LFA-1 and ICAM-1^21^, vimentin^13^, microtubule^22^ and actomyosin genes^8^,^23^. And most of them had been implicated in human tumors, suggesting a close correlation of CICs with tumor development and progression.

In this work, we report the involvement of α-catenin, a molecular linker between AJs and cytoskeleton, in controlling CICs formation of human tumor cells. Alpha-catenin is well known as a tumor suppressor, whose inactivation by multiple mechanisms contributes to advanced phenotypes of various human tumors^24^,^25^,^26^. Multiple oncogenic pathways such as YAP and NF-κb signaling were found affected by α-catenin^27^,^28^, the growth of tumor cells was usually inhibited by α-catenin in cell-autonomous way, which was confirmed in our experiment (data not shown). As formation of CICs would eventually lead to death of internalized cells^8^, therefore, it is conceivable that inactivating α-catenin could also promote tumor growth in a non-autonomous way by escaping CICs-mediated cell death. Unfortunately, due to technical issue, it is currently hard to differentiate the effects of CICs induction from other tumor inhibitory effects imposed by α-catenin, on which a cleaner cell model is expected.

Different from β-catenin, α-catenin was thought to bind cadherin complex in a dynamic fashion. Alpha-catenin could exist in form of either monomer or homo-dimmer in cells, monomer α-catenin preferentially binds β-catenin to contribute AJs formation, whereas the dimmer binds F-actin, where it inhibits branching and promotes bundling of F-actin^3^. Consistent with this model, we did observe enhanced F-actin in tumor cells upon α-catenin expression (Fig. 6), which may be attributed to excessive amount of α-catenin by forced overexpression. As a result, in addition to restoring AJs, excessive α-catenin forms homo-dimmer to promote F-actin bundling, which was thought to reduce actin dynamics^29^,^30^. The increased bundling and reduced dynamics of F-actin by α-catenin might explain the phenotype that CICs formation in H820 and MDA-MB-468 cells occurred in a relative slower pace as compared with MCF10A and MCF7 cells (Figs 1A and 3B), as active remodeling of actin cytoskeleton is required for efficient CICs formation^9^,^16^.

It should be noted that, although α-catenin was not expressed and AJs was compromised, MDA-MB-468 cells could still form CICs at low level after long time induction, suggesting that additional mechanisms existing to promote CICs formation. There are three possibilities, first, other cell-cell adhesions other than AJs could mediate weak cell-cell interaction and induce CICs at low rate, one candidate is Nectin/Afadin adhesion complex^31^; second, live cell phagocytosis in addition to active invasion plays a role in CICs formation as phagocytosis can take place independent of AJs and result in similar CICs morphology^32^; third, some other molecules could compensate α-catenin’s function in CICs formation, for example, vinculin and the like^17^. Since few CICs were formed in H820 cells in the absence of α-catenin, future work comparing the molecular differences between these two cells may help shed light on this puzzle. Meanwhile, although α-catenin expresses in some tumor cells such as MDA-MB-436, ZR75-1 and H441 (Fig. 1), these cells display low level of CICs formation upon induction, which suggests that mechanisms other than lacking α-catenin expression are responsible for CICs inhibition. Further investigation on these cells would shed light on the underpinning mechanisms in the future.

## Materials and Methods

### Cell culture

Cell line MDA-MB-468 was purchased from Beijing Union Cell Resource Center; MCF7 and MCF10A cells were from Dr. Michael Overholtzer (Memorial Sloan-Kettering Cancer Center); MDA-MB-436 and ZR75-1 cells were from Dr. Qinong Ye (Beijing Institute of Biotechnology). Breast cancer cells MCF7, MDA-MB-468, MDA-MB-436 and ZR75-1 were cultured in DMEM (Hyclone) supplemented with 10% fetal bovine serum (Gibco). Lung cancer cells H820 and H441 were kindly gifts from Dr. Ji-shuai Zhang in this institute, and were maintained in RPMI-1640 (Hyclone) with 10% fetal bovine serum. MCF10A cells were grown as described^9^ in DMEM/F12 (Hyclone) supplemented with 5% equine serum, 20 ng/ml EGF, 10 ug/ml insulin, 0.5 ug/ml hydrocortisone, and 100 ng/ml cholera toxin.

### Antibodies

Antibodies were used as follows: anti-E-cadherin (1:1000 for western blot and 1:200 for IF; BD Biosciences); anti-α-catenin (1:1000 for western blot and 1:200 for IF; BD Biosciences); β-catenin (1:1000; BD Biosciences); γ-catenin (1:1000; BD Biosciences); α-tubulin (1:4000 for western blot and 1:200 for IF; Cell Signaling); β-actin (1:1000; Cell Signaling); phalloidin (1:200; Invitrogen). All of the secondary antibodies (1:3000 for western blot and 1:300 for IF) were purchased from CWBIO, China. ROCK inhibitor Y27632 was purchased from TOCRIS and used at final concentration of 10 uM. Prolong Gold antifade reagent with DAPI was purchased from Life technologies.

### Plasmid construction

pEGFP-C1-α-catenin was a gift from W. James Nelson from Stanford University. The retroviral construct pQCXIP-EGFP-α-catenin was made by subcloning EGFP-α-catenin fusion gene from pEGFP-C1-α-catenin in between *Age* I and *Mfe* I sites of pQCXIP-EGFP-N1, which was constructed by replacing gene encoding GFP in pQCXIP-GFP-C1 with EGFP gene from pEGFP-N1. pQCXIP-GFP-C1 was gifted by Dr. Alan Hall at MSKCC. Retroviruses were made and used to infect target cells as described^23^. Virus-infected MDA-MB-468 and H820 cells were selected with puromycin at 2 ug/ml for MDA-MB-468 and 0.8 ug/ml for H820.

### siRNA transfection

MCF10A cells of 2.5 × 10^5^ were cultured in 6-well plates for 16 h before transfected with 50 uM siRNAs for α-catenin or control sequence (synthesized by GenePharma, China). The knockdown efficiency was measured by Western blot 48 h after transfection.

### Cell cluster assays

In order to detect the cell clustering degree, 2.5 × 10^5^ MDA-MB-468 or H820 cells were cultured in 6-well plates, which were pre-coated with 0.5% soft agar to prevent cell attachment. After 36 hours, cytospins were made and stained with DAPI, based which cells involved in the formation of cell cluster were quantified. Cells in cluster rate (%) = (number of tumor cells involved in cell cluster / number of total tumor cells counted) ×100. We defined cell cluster as a cell colony which contains more than 6 cells.

### Cell-in-cell formation assays

For cell-in-cell structures to be induced, about 2.5 × 10^5^ cells were cultured in 6-well plates coated with 0.5% soft agar for 6 hours or longer as indicated. Cytospins were then made by centrifugation at 1400 rpm for 5 min (Low speed tabletop centrifuge DT5-6, ERA BEILI CENTRIFUGE CO. China.) for immunostaining as described^18^ to quantify cell-in-cell structures. Internalized cells wrapped at least half-way around by outer cells were considered to be CICs.

### Immunostaining and immunoblotting

Cytospins were first fixed in 10% trichloroacetic acid or 4% paraformaldehyde for 10 min and then washed by PBS for three times, followed by immunostaining with indicated antibodies. Nuclei were counterstained with DAPI when slides were mounted with cover slips by Prolong Gold Antifade reagent for image capture after 24 h air-drying. Images were taken at three channels (FITC, TRITC and DAPI) by confocal microscopy. Western blot was performed as described^33^. Briefly, protein samples were subjected to SDS-PAGE and then transferred onto nitrocellulose membrane, after which appropriate antibodies were used to probe specified protein.

### Epi and confocal microscopy

Images of large field were taken by Nikon ECLIPSE Ti-U epi-fluorescence microscope by 10×objectives, and analysed by NIS-Elements F 3.0 software (Nikon, Japan). Besides, images of high resolution were acquired by using OLYMPUS FLUOVIEW FV1000 Confocal Microscope at three channels (FITC, TRITC, DAPI), and analyzed by FV10-ASW 4.0 Viewer software (Olympus, Japan).

### Statistics

All the experiments were carried out at least three times. Results were displayed as mean ± SD. *P* values were calculated by Two-tailed Student’s *t*-test using SPSS20.0 software (IBM), with statistical significance assumed at *P* < 0.05.

## Additional Information

**How to cite this article**: Wang, M. *et al.* Impaired formation of homotypic cell-in-cell structures in human tumor cells lacking alpha-catenin expression. *Sci. Rep.* **5**, 12223; doi: 10.1038/srep12223 (2015).
